# Research priorities for improving menstrual health across the life-course in low- and middle-income countries

**DOI:** 10.1080/16549716.2023.2279396

**Published:** 2023-11-27

**Authors:** Marina Plesons, Belen Torondel, Bethany A. Caruso, Julie Hennegan, Marni Sommer, Jacquelyn Haver, Danielle Keiser, Anna M. van Eijk, Garazi Zulaika, Linda Mason, Penelope A. Phillips-Howard

**Affiliations:** a Department of Public Health Sciences, Miller School of Medicine, University of Miami, Miami, FL, USA; b Department of Infectious Diseases, London School of Hygiene and Tropical Medicine, London, UK; c Hubert Department of Global Health, Rollins School of Public Health, Emory University, Atlanta, GA, USA; d Maternal, Child, and Adolescent Health Program, Burnet Institute, Melbourne, Australia; e Department of Sociomedical Sciences, Mailman School of Public Health, Columbia University, New York, NY, USA; f School Health and Nutrition, Department of Education and Children Protection, Save the Children US, Washington, DC, USA; g Research Unit, Menstrual Health Hub, Berlin, Germany; h Department of Clinical Sciences, Liverpool School of Tropical Medicine, Liverpool, UK

**Keywords:** Menstrual hygiene management, menstrual health, WASH, Education, gender equity

## Abstract

**Background:**

Research on menstrual health is required to understand menstrual needs and generate solutions to improve health, wellbeing, and productivity. The identification of research priorities will help inform where to invest efforts and resources.

**Objectives:**

To identify research priorities for menstrual health across the life-course, in consultation with a range of stakeholder groups from a variety of geographic regions, and to identify if menstrual health research priorities varied by expertise.

**Methods:**

A modified version of the Child Health and Nutrition Research Initiative approach was utilized to reach consensus on a set of research priorities. Multisector stakeholders with menstrual health expertise, identified through networks and the literature, were invited to submit research questions through an online survey. Responses were consolidated, and individuals were invited to rank these questions based on novelty, potential for intervention, and importance/impact. Research priority scores were calculated and evaluated by participants’ characteristics.

**Results:**

Eighty-two participants proposed 1135 research questions, which were consolidated into 94 unique research questions. The mean number of questions did not differ between low- and middle-income country (LMIC) and high-income country (HIC) participants, but significantly more questions were raised by participants with expertise in mental health and WASH. Sixty-six participants then ranked these questions. The top ten-ranked research questions included four on ‘understanding the problem’, four on ‘designing and implementing interventions’, one on ‘integrating and scaling up’, and one on ‘measurement’. Indicators for the measurement of adequate menstrual health over time was ranked the highest priority by all stakeholders. Top ten-ranked research questions differed between academics and non-academics, and between participants from HICs and LMICs, reflecting differences in needs and knowledge gaps.

**Conclusions:**

A list of ranked research priorities was generated through a consultative process with stakeholders across LMICs and HICs which can inform where to invest efforts and resources.

## Background

Menstrual health has received increased attention in recent years as an important component of public health [1,2]. Research in low- and middle-income countries (LMICs) – largely descriptive studies – has described girls’ need for information related to menstrual health and the impacts of poor menstrual health on their health, wellbeing, and education [3–5]. A small number of recent trials have evaluated the impact of menstrual products and puberty education on girls’ school attendance, educational performance, sexual and reproductive health (SRH), and wellbeing [6–12]. Other studies have focused on understanding the menstrual self-care practices and menstrual health challenges of women and girls in humanitarian contexts, as well as the acceptability of specific menstrual products [13–15] and policy considerations [16] in such settings. More recently, studies have started to describe challenges for adult women [17–21] and marginalized populations [22–27], the relationship between menstrual health and mental health [28], and measures for menstrual health research [5,29–31]. The evidence on menstrual health has been consolidated into a growing body of systematic reviews, including reviews focused on specific geographies [32–34], populations (e.g. girls with disabilities [35], and those who are displaced [32,36]), measures of exposures and outcomes [30,37], interventions (e.g. menstrual cups, reusable menstrual pads) [38,39], and outcomes (e.g. knowledge and understanding, health, and social wellbeing) [4,34,40–42].

The identification of research priorities is an important process to help researchers, programmers, practitioners, policymakers, and funding agencies decide on which specific areas to invest their efforts and resources. In 2014, research priorities on menstrual hygiene management (MHM) among schoolgirls in LMICs were identified by an expert group as part of ‘MHM in Ten,’ an initiative that sought to set the agenda for overcoming challenges related to menstrual health and hygiene (MHH) faced by this population and for identifying the evidence needed to improve girls’ experiences of menstruation and education [1,3]. As evidenced by its extensive citation history and the substantial number of relevant research outputs since 2014, it is clear that the prioritization effort has positively impacted the trajectory of the field, which has developed rapidly in the past nine years. Several identified priorities have been acted upon, including the need to strengthen the evidence base, with over 50% of all menstrual health literature published after 2015 [4]. Other priorities have also seen progress, including the need for standardized menstrual measures [31,43] and definitions [30,44,45], and the need for a research consortium, which has begun to take shape under the umbrella of the Global Menstrual Collective (GMC). The GMC is a collaborative network whose aim is to bring together partners in MHH to amplify efforts and reduce duplication for mainstreaming menstrual health across health, education, gender, and water, sanitation, and hygiene (WASH).

Given the evolution of the field, it is therefore timely to reassess the research priorities for improving menstrual health to guide the field. Further, as the focus of efforts to address menstrual health has expanded beyond schoolgirls to include girls who are out-of-school, as well as women and others who menstruate, there is a need to identify research priorities to address the needs of all who menstruate across the life-course in varying contexts around the world [46]. According to the WHO, a life course approach to health aims to ensure people’s well-being at all ages by addressing people’s needs, ensuring access to health services, and safeguarding the human right to health throughout their lifetime [47].

Thus, the objective of this study is to identify research priorities for menstrual health across the life-course in LMICs, in consultation with a range of stakeholder groups from a variety of geographic regions. This study additionally aims to understand if and how research priorities differ across sectors, specifically between academics and those outside of academia.

## Methods

This research was undertaken by members of the Global Menstrual Collective’s Research and Evidence Group, comprising researchers, consultants, programmers, policymakers, and funding agencies working in the field of menstrual health.

We used a modified version of the Child Health and Nutrition Research Initiative (CHNRI) approach to reach consensus on a set of research priority questions. The CHNRI approach is a transparent and structured process for ranking the relative importance of competing research priorities to help decision-makers effectively allocate limited resources to address a health problem, e.g. by reducing morbidity and mortality, improving wellbeing and quality of life, and addressing inequities [48,49]. It has been used to reach consensus on research priorities for numerous health topics, including adolescent health [50], adolescent sexual and reproductive health [51], and family planning [52]. In short, the CHNRI approach involves three phases: 1) identifying individuals with expertise on a topic; 2) asking these individuals to propose research questions related to the topic; and 3) asking them to rate the proposed research questions against a set of criteria. These ratings are subsequently used to calculate a composite Research Priority Score for each question, which are then ranked. The adapted CHNRI approach used in this study is described in detail below.

### Phase 1: identification of individuals with expertise in menstrual health

To be as inclusive as possible, a snowball and self-selection approach was used for this study. Phase I consisted of identifying ways of reaching individuals from various stakeholder groups with expertise in policy, programming, financial support, and/or research related to menstrual health around the world. This included (i) menstrual health networks, coalitions, and consortia (e.g. the Menstrual Health Hub, the African Coalition for Menstrual Health Management, the MHM in Ten Expert network, Water Supply and Sanitation Collaborative Council and the GMC Collective, the Menstrual Cup Collaboration, and WASH United’s Menstrual Hygiene Day), (ii) published researchers, and (iii) funders from past or current funding calls.

### Phase 2: identification of research questions on menstrual health

An initial correspondence was sent to (i) menstrual health networks, coalitions, and consortia, (ii) known academic researchers likely to be missed from (i), and (iii) funders identified in Phase 1. This correspondence included information about the study, an invitation to participate, and an electronic survey (SurveyMonkey, Palo Alto) through which they could propose research questions regarding menstrual health across the life-course. The invitation email was sent in September 2020 with two reminders sent fortnightly. The survey was closed in October 2020.

Participants were prompted to propose research questions after they read an information sheet explaining the nature and purpose of the exercise, they had consented, and had provided demographic information about their sector of work (e.g. academia, UN-agency, non-governmental organization) and geographic areas of residence and work Research questions spanned three domains (each with several sub-domains), as guided by the CHNRI approach:

1. *Understanding the problem*: questions to illustrate the experiences of those who menstruate, explore risk and protective factors for menstrual health, and test impacts and consequences of poor menstrual health. Such questions could utilize a range of methodologies, from descriptive epidemiology to ethnographic research.
2. *Designing and implementing interventions*: questions which relate to (i) discovery of new interventions, (ii) development and testing the effectiveness of interventions, (iii) evaluations of the costs of interventions, (iv) evaluations of the delivery of interventions (including acceptability, adoption, appropriateness, feasibility, fidelity, coverage, and reach), and (v) evaluations of the sustainability of interventions. Such questions could utilize intervention effectiveness research and implementation research.
3. *Integrating and scaling up interventions*: questions which relate to integrating menstrual health interventions into health, education, WASH, or social services and to taking menstrual health interventions to scale. Such questions could include implementation research and policy and systems research.

Exemplar questions for each domain and sub-domain were included in the survey to provide further clarity for the participants.

Participant responses were downloaded into spreadsheets, and free texts were collapsed to aggregate group data. A core team of four members of the Global Menstrual Collective’s Research and Evidence Group then iteratively categorized and consolidated the questions based on themes. Further ordering of questions between domains and sub-domains was undertaken where relevant. Duplicates were removed, as were questions covering unrelated topics. An extra domain, *Measurement & Research*, was included as numerous questions on this topic were suggested by participants. Similar questions were condensed together to derive a smaller number of amalgamated research questions (Table S1). Once the full set of consolidated questions was developed, a meeting with the GMC’s Research and Evidence Group was held to review and agree upon a final list of research questions to be used in Phase 3.

### Phase 3: prioritization of the proposed research questions on menstrual health

Following the CHNRI approach, Phase 3 of the process involved rating each of the proposed research questions on a standard set of criteria to generate a composite Research Priority Score for each question. Discussion among the GMC’s Research and Evidence Group raised concerns about the length of the survey that would be required to accomplish this, given the large number of research questions that were proposed by participants in Phase 2. Further, the relevance of the five criteria typically proposed by the CHNRI approach (i.e. clarity, answerability, importance/impact, implementation, equity) was questioned for this specific topic; in particular, there was concern for potential confounding due to the explicit mention of ‘equity’ in multiple questions. To address these concerns, the five criteria were modified in line with the CHNRI approach, which suggests that the priority setting process should list possible criteria appropriate to their specific context and may merge criteria, where appropriate [49]. Thus, three criteria – novelty, potential for implementation, and importance/impact (Table 1) – were agreed upon, with the CHNRI approach’s standard scoring system of *yes, no, or undecided*. Due to its length, the survey was split into two sections comprising 43 and 51 research questions.

**Table 1.** Criteria used to score the proposed research questions and their definitions.

	Understanding the problem	Designing and implementing interventions	Integrating and scaling-up interventions	Measurement & research
Novelty	Will the answer to this question fill a key gap?			
Potential for implementation	Will the answer to the question contribute to tailored interventions?	Will the answer to the question result in an intervention that can be implemented?	Could the answer to the question be implemented?	
Importance/impact	Will the answer to this question be important to know?	Will the answer to the question result in an intervention that would have an important impact?	Will the answer to the question have an important impact?	

Invitations were sent to (i) menstrual health networks, coalitions, and consortia, (ii) published researchers, and (iii) funders identified in Phase 1 to score the proposed research questions, again using an electronic survey (SurveyMonkey, Palo Alto). An invitation email was sent in June 2021 with two reminders sent fortnightly. The survey was closed in July 2021. Similar to the prior survey, participants read the information sheet, consented, and provided information on their sector and area of work.

Participant responses were downloaded into spreadsheets, cleaned, and imported into IBM SPSS version 28 (Armonk, N.Y.). A variable was created to indicate if participants had responded to individual research priority questions. The number of responses per participant and per research question were counted. For participant demographic information, frequency distributions of characteristics of the participants were conducted. We also conducted an analysis of variance to test if there was a significant difference between the mean number of questions, across the three domains and overall, provided by participants with seven or more years of experience with MHH compared with those with less experience. Means, standard deviations, and levels of significance reached were generated, with a p-value of ≤ 0.05 considered significant.

For ranking of the research questions, a score of 100 points was attributed to ‘yes’, 50 points to ‘undecided’, and 0 points to ‘no’. A total Research Priority Score (RPS) was then assigned for each research question by computing the mean score across the three criteria. RPSs were then ranked from highest to lowest, overall and within each domain. RPSs were also assessed based on the profile of the participants, e.g. by their sector of work, stakeholder group, and years working in menstrual health.

### Ethical considerations

The project was approved by the Liverpool School of Tropical Medicine’s Research and Ethics Committee (ID# 20–055), and it was granted exemption from review by the Human Reproduction Programme Research Protocol Review Panel and the WHO Ethics Review Committee (ID# ERC.0003407). Potential participants were informed that their participation was voluntary, and they were free to stop responding to the questions at any time. Participants were required to indicate their consent using a checkbox before the survey commenced.

## Results

### Characteristics of phase 2 participants

A total of 82 participants responded to the Phase 2 survey and proposed research questions on menstrual health (Table 2). The majority were female (89%) with 33% and 28% aged 25–34 years and 35–44 years respectively. The highest proportion (*N* = 50, 61%) originated from HICs, with 29% from Europe; LMICs were represented by 30 (39%) of participants, with 27% of all participants from sub-Saharan Africa. The highest proportion of participants worked in non-government organization (NGOs) and international NGOs (43%) or in academia (35%). A higher proportion of participants from HIC were academics, compared with LMIC (48% versus 15.6%, *p* = 0.002), while the reverse was true for NGOs, where 34% were from LMIC and 16.0% from HIC (*p* = 0.05). A third of participants worked globally, over half (58%) worked in sub-Saharan Africa, and 35% worked in east and southern Asia. Sixty-two percent of participants reported their area of expertise lay in sexual and reproductive health (SRH). Over one third (38%) of participants had worked in the field of menstrual health for seven years or longer.

**Table 2.** Participant characteristics (*n* = 82).

		Participants of Phase 2 (*n* = 82)	Participants of Phase 3 (*n* = 66)
Age	18–24 25–34 35–44 45–54 55–64 65+	4 (4.9%) 27 (32.9%) 23 (28.0%) 15 (18.3%) 7 (8.5%) 6 (7.3%)	0 17 (25.8%) 16 (24.2%) 18 (27.3%) 6 (9.1%) 8 (12.1%)
Gender	Female Male Non-binary or gender not listed above	73 (89.0%) 9 (11.0%) 0	53 (80.3%) 12 (18.2%) 1 (1.5%)
Region of world where the participants reside	Europe North America Sub-Saharan Africa Asia Australasia Middle East Latin America	24 (29.3%) 19 (22.2%) 22 (26.8%) 8 (9.8%) 7 (8.5%) 2 (2.4%) 0 (0%)	21 (31.8%) 17 (25.8%) 11 (16.7%) 12 (18.2%) 2 (3.0%) 0 (0%) 3 (4.5%)
High or low-middle income country residence	HIC LMIC	50 (61.0%) 32 (39.0%)	40 (60.6%) 26 (39.4%)
Sector of work	Academia NGO International NGO International Agency Entrepreneur Funding Agency Government Other	29 (35.4%) 19 (23.2%) 16 (19.5%) 10 (12.2%) 7 (8.5%) 2 (2.4%) 2 (2.4%) 12 (14.6%)	22 (33.3%) 14 (21.2%) 11 (16.7%) 7(10.6%) 6 (9.1%) 7 (10.6%) 4 (6.1%) 7 (10.6%)
Region of world where the participants work	Sub-Saharan Africa Global Asia North America Europe Pacific Middle-East Latin America North Africa	38 (58.5%) 30 (36.6%) 29 (35.4%) 12 (14.6%) 12 (14.6%) 11 (13.4%) 8 (9.8%) 7 (8.5%) 7 (8.5%)	25 (37.9%) 26 (39.4%) 20 (30.3%) 11 (16.7%) 12 (18.2%) 6 (9.1%) 0 (0%) 8 (12.1%) 0 (0%)
Areas of expertise*	SRH Adolescent Health/Development WASH Gender Policy/Advocacy Education Mental Health Other	51 (62.2%) 39 (47.6%) 35 (42.7%) 32 (39.0%) 30 (35.6%) 26 (31.7%) 11 (13.4%) 17 (20.7%)	43 (65.2%) 33 (50.0%) 27 (40.9%) 24 (36.4%) 21 (31.8%) 20 (30.3%) 6 (9.1%) 11 (16.7%)
Years of experience working on menstrual health	<12 m 1-3y 4-6y 7-9y 10y+	5 (6.1%) 18 (22.0%) 28 (34.1%) 16 (19.5%) 15 (18.3%)	1 (1.5%) 11 (16.7%) 19 (28.8%) 12 (18.2%) 22 (33.3%)

*More than one area of expertise could be answered.

### Proposed research questions

A total of 1135 research questions were proposed by the 82 participants that responded to the Phase 2 survey, with a mean of 13.8 (standard deviation [sd 9.9]; median 11) research questions proposed per participant (Table 3 and Table S2). The greatest number of research questions proposed were on ‘understanding the problem’ (521 by all 82 participants; mean 6.4 per participant), followed by ‘designing and implementing interventions’ (451 by 69 participants, mean 6.5 per participant). Integrating and scale up questions were proposed by fewer participants (145 questions, among 54 participants, mean 2.7), and 15 participants suggested 18 other research questions (mean 1.2). Participants with seven or more years of experience in menstrual health (*n* = 51) proposed more research questions (average 17.6), compared with those with fewer years of experience (*n* = 31; average 11.7) (Table S3). Participants with WASH expertise (*N* = 35) provided significantly more questions (16.77, sd 9.72) compared with those without WASH expertise (*N* = 47; 11.85, sd 9.63) (Table S4). Participants working in the mental health sector (*N* = 11) also proposed more research questions (mean 19.7, sd 12.9) than those working in other areas (combined mean 13.17, sd 9.25). The differences among stakeholder groups were less pronounced, with no significant differences. Evaluation of the research priority questions posed by LMIC (*N* = 32) compared with HIC (*N* = 50) participants found no significant differences in the mean number of research questions, with a mean of 15.03 (sd 9.82) suggested by LMIC participants compared with a mean of 13.29 (sd 10.02; *p* = 0.442) from HIC participants.

**Table 3.** Overview of the proposed research questions.

Domain	Total number of research questions proposed	Number of participants that proposed at least one research question	Average number of research questions proposed per participant
Understanding the problem	521 (45.9%)	82	6.4
Designing and implementing interventions	451 (39.7%)	69	6.5
Integrating and scale-up	145 (12.7%)	54	2.7
Other	18 (1.6%)	15	1.2
**Total**	**1135**		**13.8**

As previously described, the proposed research questions were consolidated into a final list of 94 unique research questions. A breakdown of the number of research questions per domain and sub-domain is provided in Table 4.

**Table 4.** Overview of the consolidated list of research questions.

Domain	Sub-domain	Number of research questions
Understanding the problem	Experiences related to menstrual health	7
	Factors affecting menstrual health	10
	Impact and consequences	8
	**Total**	**25**
Designing and implementing interventions	Discovery of new interventions	12
	Developing and testing the effectiveness of interventions/programmes	19
	Evaluations of the costs of interventions/programmes	6
	Evaluations of the delivery (including acceptability, adoption, appropriateness, feasibility, fidelity, coverage, and reach) of interventions/programmes	10
	Evaluations of the sustainability of interventions/programmes	6
	**Total**	**53**
Integrating and scale-up	Integration	6
	Scale-up	5
	**Total**	**11**
Measurement & Research		**5**
**Grand Total**		**94**

### Characteristics of phase 3 participants

A total of 66 participants completed the Phase 3 survey, contributing to the prioritization of the proposed research questions (Table 2). In the interest of ensuring anonymity of the data, it was not possible to confirm that the participants who responded to the survey in Phase 2 were the same as those who responded to the survey in Phase 3. The majority were female (80%), with 26% and 27% aged 25–34 years and 45–54 years respectively. The highest proportion (61%) resided in HICs, with 32% of the participants from Europe; LMICs were represented by 39%, with 18% of the participants from Asia. The highest proportion of participants worked in NGOs or international NGOs (38%) and academia (33%). An equal proportion of participants from LMIC and HIC were academics (34.6% and 32.5%, respectively). Over a third (39%) of participants worked globally, 38% worked in sub-Saharan Africa, and 30% worked in east and south Asia. Sixty-five percent of participants reported working in SRH. Over half (51%) had worked in the field of menstrual health for seven years or more.

### Prioritized research questions

The highest ranked research question by RPS among all participants was ‘What indicators are optimal for assessing menstrual health over time (e.g. related to norms, education, health, rights, etc.)?’ It was ranked highest according to non-academic participants, and second highest according to academic participants.

**Figure 1.**
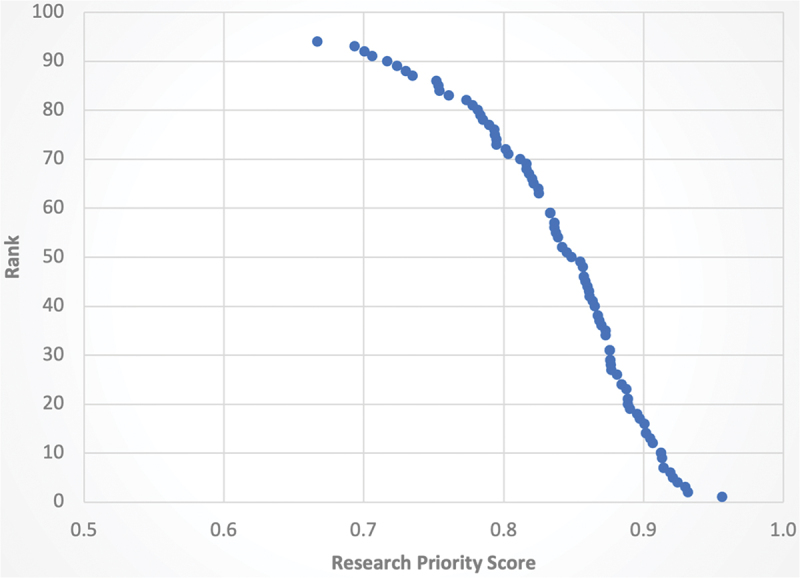
Research priority scores and overall rank.

The full list of ranked research questions is available in Table S5. The top ten-ranked research questions are listed in Table 5. They include four questions on ‘understanding the problem’, four on ‘designing and implementing interventions’, one on ‘integrating and scaling up’, and one on ‘measurement and research’. We found a high level of agreement on these ten questions, with total RPS ranging from 0.913 to 0.956 (out of a possible 1). Of note, the difference in RPS between the top ten-ranked research questions and those subsequent was not substantial, pointing to the high prioritization of many questions beyond those included only in this abbreviated list (Figure 1). Thus, the top five-ranked research questions in each domain, ranked according to their RPS, are listed in Table 6.

**Table 5.** Top 10-ranked research questions, by research priority score.

Rank	RPS	Question	Domain	Unique ID
1	0.956	What indicators are optimal for assessing menstrual health over time (e.g. related to norms, education, health, rights, etc.)?	Measurement and research	M1
2	0.932	What are the experiences of girls, women, and others who menstruate in relation to menstrual pain and disorders (e.g. what proportion experience them, what are their perceptions about them, how do they manage them, and what support do they seek and receive for them)?	Understanding the problem	U1
3	0.93	What new interventions could be developed to address harmful attitudes, norms and stigma and improve communication related to menstruation?	Designing and implementing interventions	D1
4	0.924	Are girls, women, and others who menstruate able to access and afford their preferred menstrual product/materials; what is the quality of these products/materials; where do they obtain them; and how do they use and dispose of them?	Understanding the problem	U2
5	0.921	What is the impact of unmet menstrual health needs (e.g. products/materials, WASH infrastructure/services) on the participation and engagement of girls, women, and others who menstruate in school and work and their self-esteem and agency?	Understanding the problem	U3
6	0.919	What are the experiences and challenges of girls, women, and others who menstruate with particular needs and circumstances (e.g. those living with HIV, those with disabilities, those who are incarcerated, those experiencing homelessness, those who have experienced FGM, trans and gender non-binary persons) in relation to their menstrual health?	Understanding the problem	U4
7	0.915	What characteristics of menstrual health interventions/programmes enable them to be sustained over time?	Designing and implementing interventions	D2
7	0.915	How can interventions to address menstrual health (e.g. education, social norm change, distribution of menstrual products/materials, improvements in WASH infrastructure/services, provision of health services for menstrual pain and disorders) be scaled up with quality and equity?	Integrating and scaling up	I1
9	0.913	What new interventions could be developed to manage menstrual pain?	Designing and implementing interventions	D3
10	0.913	What are the impacts of unconditional and conditional cash transfer interventions on the menstrual health of girls, women, and others who menstruate, and consequently on their education, work, and social participation?	Designing and implementing interventions	D4

**Table 6.** Top five-ranked research questions in each domain, by research priority score.

Domain	Rank within domain	Overall rank	RPS	Question	Unique ID
Understanding the problem (*n* = 25)	1	2	0.932	What are the experiences of girls, women, and others who menstruate in relation to menstrual pain and disorders (e.g. what proportion experience them, what are their perceptions about them, how do they manage them, and what support do they seek and receive for them)?	U1
	2	4	0.924	Are girls, women, and others who menstruate able to access and afford their preferred menstrual product/materials; what is the quality of these products/materials; where do they obtain them; and how do they use and dispose of them?	U2
	3	5	0.921	What is the impact of unmet menstrual health needs (e.g. products/materials, WASH infrastructure/services) on the participation and engagement of girls, women, and others who menstruate in school and work and their self-esteem and agency?	U3
	4	6	0.919	What are the experiences and challenges of girls, women, and others who menstruate with particular needs and circumstances (e.g. those living with HIV, those with disabilities, those who are incarcerated, those experiencing homelessness, those who have experienced FGM, trans and gender non-binary persons) in relation to their menstrual health?	U4
	5	18	0.895	How do financial barriers impact the ability of girls, women, and others who menstruate to manage their menstruation?	U5
Designing and implementing interventions (*n* = 53)	1	3	0.93	What new interventions could be developed to address harmful attitudes, norms and stigma and improve communication related to menstruation?	D1
	2	7	0.915	What characteristics of menstrual health interventions/programmes enable them to be sustained over time?	D2
	3	9	0.913	What new interventions could be developed to manage menstrual pain?	D3
	4	10	0.913	What are the impacts of unconditional and conditional cash transfer interventions on the menstrual health of girls, women, and others who menstruate, and consequently on their education, work, and social participation?	D4
	5	11	0.913	What are the impacts of providing free/subsidized menstrual products/materials on menstrual health?	D5
Integrating and scaling up (*n* = 11)	1	8	0.915	How can interventions to address menstrual health (e.g. education, social norm change, distribution of menstrual products/materials, improvements in WASH infrastructure/services, provision of health services for menstrual pain and disorders) be scaled up with quality and equity?	I1
	2	14	0.902	How can information on menstruation be integrated into existing formal and non-formal educational curricula, health services (e.g. contraceptive services, HPV vaccination, FGM support, psychosocial support), social norms, and gender equality interventions/programmes?	I2
	3	15	0.902	How can considerations for the menstrual health needs of girls, women, and others who menstruate with particular needs and circumstances (e.g. those with disabilities and their caregivers, those living in urban/rural settings, those who have experienced FGM, those in indigenous communities, those in humanitarian settings) be integrated into existing policies and interventions/programmes?	I3
	4	17	0.897	How can governments integrate menstrual health across sectors (e.g. education, health, WASH, gender) and achieve multi-sectoral coordination?	I4
	5	32	0.876	How can integration of menstrual health across sectors be optimally monitored and evaluated?	I5
Measurement and research (*n* = 5)	1	1	0.956	What indicators are optimal for assessing menstrual health over time (e.g. related to norms, education, health, rights, etc.)?	M1
	2	19	0.890	What tools/instruments/approaches/measures are optimal for assessing the impact of interventions to address menstrual health at various programmatic levels (e.g. local, national, global)?	M2
	3	37	0.868	What is the definition of a meaningful improvement in menstrual health, and how can it be measured?	M3
	4	78	0.785	How can lessons learned from the delivery of menstrual health interventions be optimally documented and shared?	M4
	5	89	0.724	What support is needed to encourage relevant, timely, and rigorous research on menstrual health, and which actors need to be engaged in the research process?	M5

When examining the top five-ranked research questions by individual scoring criteria, as opposed to the overall RPS, it is notable that the top five-ranked questions in the criteria ‘potential for implementation’ are in the overall top ten ranked questions by RPS, as are the top five ranked questions in the criteria ‘importance/impact’, except for one question (Table 7). In both criteria, three out of the top five-ranked questions are in the domain ‘understanding the problem.’ Additionally, it is noteworthy that the top five-ranked questions in the criteria ‘novelty’ did not include questions from the domain on ‘understanding the problem’ and instead included questions from the domains of ‘designing and implementing interventions’, ‘integration and scale-up’, and ‘measurement and research.’

**Table 7.** Top five research questions ranked by individual scoring criteria.

Individual scoring criteria	Rank within criteria	Overall rank	Question	Overall RPS	Unique ID
Novelty	1	16	How long should menstrual cups be worn, how should they be cleaned between use, and how often should they be replaced?	0.901	D8
	2	17	How can governments integrate menstrual health across sectors (e.g. education, health, WASH, gender) and achieve multi-sectoral coordination?	0.897	I4
	3	1	What indicators are optimal for assessing menstrual health over time (e.g. related to norms, education, health, rights, etc.)?	0.956	M1
	4	6	What new interventions could be developed to manage menstrual pain?	0.913	D3
	5	10	What are the impacts of providing free/subsidized menstrual products/materials on menstrual health?	0.913	D5
Potential for implementation	1	1	What indicators are optimal for assessing menstrual health over time (e.g. related to norms, education, health, rights, etc.)?	0.956	M1
	2	4	Are girls, women, and others who menstruate able to access and afford their preferred menstrual product/materials; what is the quality of these products/materials; where do they obtain them; and how do they use and dispose of them?	0.924	U2
	3	3	What new interventions could be developed to address harmful attitudes, norms and stigma and improve communication related to menstruation?	0.930	D1
	4	2	What are the experiences of girls, women, and others who menstruate in relation to menstrual pain and disorders (e.g. what proportion experience them, what are their perceptions about them, how do they manage them, and what support do they seek and receive for them)?	0.932	U1
	5	6	What are the experiences and challenges of girls, women, and others who menstruate with particular needs and circumstances (e.g. those living with HIV, those with disabilities, those who are incarcerated, those experiencing homelessness, those who have experienced FGM, trans and gender non-binary persons) in relation to their menstrual health?	0.919	U4
Importance/impact	1	1	What indicators are optimal for assessing menstrual health over time (e.g. related to norms, education, health, rights, etc.)?	0.956	M1
	2	2	What are the experiences of girls, women, and others who menstruate in relation to menstrual pain and disorders (e.g. what proportion experience them, what are their perceptions about them, how do they manage them, and what support do they seek and receive for them)?	0.932	U1
	3	5	What is the impact of unmet menstrual health needs (e.g. products/materials, WASH infrastructure/services) on the participation and engagement of girls, women, and others who menstruate in school and work and their self-esteem and agency?	0.921	U3
	4	23	What is the impact of unmet menstrual health needs (e.g. information, products/materials, WASH infrastructure/services) the health and wellbeing of on girls, women, and others who menstruate across the life course?	0.888	U6
	5	3	What new interventions could be developed to address harmful attitudes, norms and stigma and improve communication related to menstruation?	0.930	D1

When examining the average RPS by domain, we found a similar level of prioritization of the domains among all participants, with average RPS ranging from 0.833 to 0.862 (Table 8). However, when comparing the average RPS by domain among academics compared with those outside of academia, it is noted that both academics and non-academics gave highest prioritization to ‘integrating and scaling up’. For academics this was followed by ‘understanding the problems’, ‘designing and implementing interventions’, and ‘measurement and research’, while for non-academics this was followed by ‘measurement and research’, ‘understanding the problem’, and ‘designing and implementing interventions’. Likewise, when comparing the average RPS by domain among participants from HICs vs LMICs, it is noted that participants from LMICs gave highest prioritization to ‘measurement and research’, followed by ‘integrating and scaling up’, ‘designing and implementing interventions’, and ‘understanding the problem’, while participants from HICs gave highest prioritization to ‘integrating and scaling up’, followed by ‘understanding the problem’, ‘measurement and research’, and ‘designing and implementing interventions’.

**Table 8.** Average research priority scores, by domain, stakeholder group, and country of origin.

Domain	Total number of research questions	Average Research Priority Scores				
		All participants	Academics	Non-academics	HIC	LMIC
Understanding the problem	25	0.850	0.843	0.853	0.843	0.860
Designing and implementing interventions	53	0.833	0.841	0.830	0.814	0.865
Integrating and scaling up	11	0.862	0.850	0.867	0.854	0.875
Measurement and research	5	0.845	0.815	0.857	0.821	0.886

**Figure 2.**
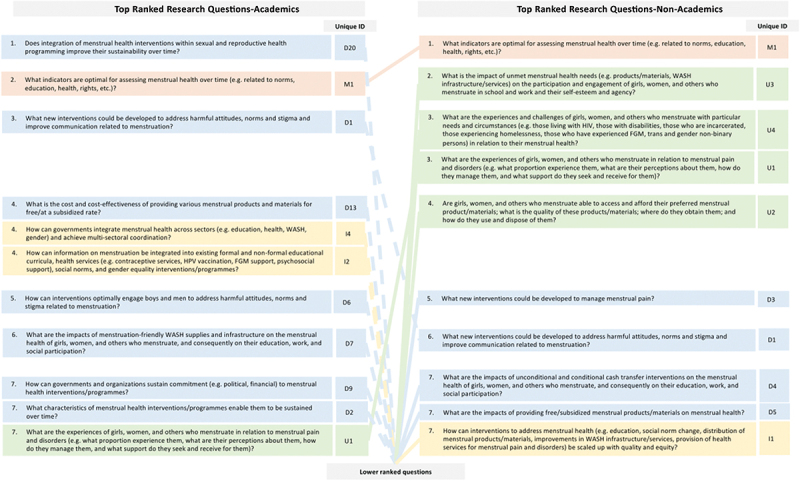
Comparison of the top ranked research questions, among academics vs. non-academic.

When comparing the top ten-ranked research questions among academics vs. those not working in academia, there were notable differences (Figure 2). First, the lists have only three questions in common (out of a total of 18 unique questions in the two lists): one regarding the optimal indicators for assessing menstrual health, one regarding interventions to address norms and attitudes about menstruation, and one regarding interventions to manage menstrual pain. Second, the top ten-ranked questions among non-academics included more questions in the domain on ‘understanding the problem’ (*n* = 4) than those among academics (*n* = 1). The top ten-ranked questions among academics, meanwhile, included more questions in the domain on ‘designing and implementing interventions’ (*n* = 7) than those among non-academics (*n* = 4).

**Figure 3.**
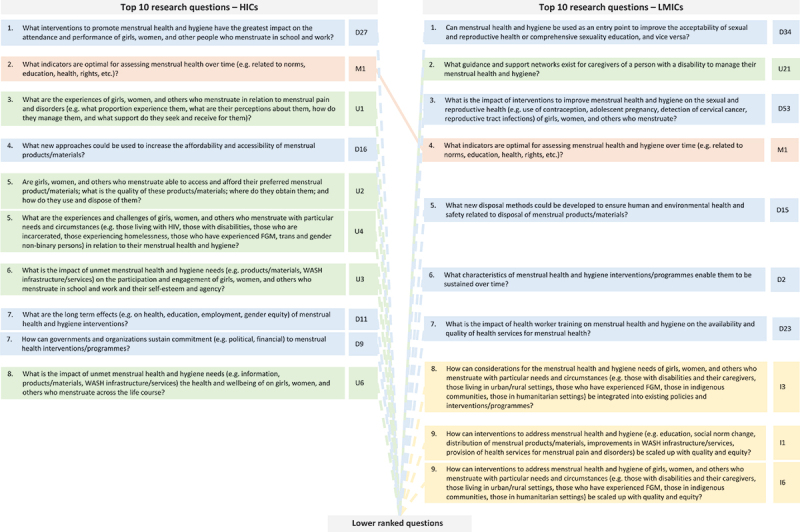
Comparison of the top ranked research questions, among participants from HICs vs. LMICs.

Finally, when comparing the top ten-ranked research questions among participants from HIC vs. LMICs, there were also considerable differences (Figure 3). First, the lists only had one question in common (out of a total of 19 unique questions in the two lists): that regarding the optimal indicators for assessing menstrual health. Second, the top ten-ranked research questions among participants from HICs included more questions in the domain on ‘understanding the problem’ (*n* = 5) than those among participants from LMICs (*n* = 1). The top ten-ranked questions among participants from LMICs meanwhile, included more questions in the domain of ‘integrating and scaling up’ (*n* = 3) than those among participants from HICs (*n* = 0).

## Discussion

This study used a modified version of the CHNRI approach to identify research priorities on menstrual health across the life-course in LMICs, moving beyond the previous research priority setting exercise conducted in 2014 that focused on schoolgirls [3]. In doing so, it incorporated input from 82 participants across all continents with expertise in policy, programming, financial support, and/or research related to menstrual health.

Overall, the study identified a greater number of research questions in the domains of ‘understanding the problem’ and ‘designing and implementing interventions’, with a higher prioritization of those research questions, compared with questions on ‘integration and scale-up’ and ‘measurement and research’. This suggests that there are still many knowledge gaps related to understanding the menstrual experiences of women, girls, and others who menstruate, and in identifying and assessing the most effective interventions to meet their needs. These two domains also align with previous research priority-setting exercises, which specifically noted ‘the need for a strong evidence base’, and included illustrative questions related to understanding the problem and developing interventions [3,53]. Until these gaps are addressed, it appears that stakeholders perceive questions regarding ‘integration and scale-up’ to be premature. The lower prioritization of questions on ‘integration and scale-up’ may also reflect the composition of stakeholder groups that participated in this study, as only a small proportion of participants represented international agencies, government, and funding agencies.

Despite there being fewer ‘measurement and research’ questions identified and prioritized overall, the top-ranked research question identified in this study was ‘What indicators are optimal for assessing menstrual health over time?’ The 2014 research priority setting exercise focused on schoolgirls also highlighted a broad need for standardized measures, and specifically noted a need for identifying indicators for national-level monitoring for assessing changes over time [3]. Having a standardized set of indicators is critical even to answer the other research questions in this list. A standardized set of indicators would allow for comparison of menstrual health issues across and within different populations worldwide, which could help researchers, implementers, and funders to target their efforts where they are needed most. Since this study was initiated, progress on indicators has been made [43]. Specifically, a shortlist of priority indicators for monitoring girls’ menstrual health at the national level [31] and a list of potential indicators for monitoring menstruation among those who work outside the home have been published [54]. Additionally, in 2021, the Joint Monitoring Programme for Water Supply, Sanitation, and Hygiene – custodians of monitoring data for SDG targets 6.1 and 6.2—included a set of harmonized menstrual health indicators as part of the first dedicated section on menstrual health in the regular reporting on household drinking water, sanitation, and hygiene [55].

This study identified that the top research priorities were not limited to one area of expertise (e.g. education, health, WASH etc.) but were distributed across topics. For example, the top ten-ranked research priorities include questions related to menstrual pain, socio-cultural drivers of menstrual health, menstrual products, and participation in school and work. This indicates that research gaps exist in multiple domains of menstrual health, which will need to be addressed through collaborative efforts across all areas of expertise. Further, they identify a strong need to promote equity by understanding the specific menstrual needs and experiences of underserved populations (e.g. those living with HIV, those with disabilities, those who are incarcerated, those experiencing homelessness, those who have experienced Female Genital Mutilation (FGM), trans and gender non-binary persons), and identifying effective interventions to meet those needs. Finally, the difference in RPS between the top 10-ranked research questions and those following was not substantial, pointing to the high prioritization of many questions beyond those included only in this abbreviated list.

This study also identified important differences in the top ten-ranked research priorities among academics and those working outside of academia, and those from HICs and LMICs. In part, the different priorities may reflect what the two stakeholder groups see firsthand as challenges and needs in their day-to-day work. It may also reflect an important need for improved knowledge sharing between stakeholder groups. For example, while sorting through the proposed research questions in Phase 2, the GMC’s Research and Evidence Group – which itself was largely composed of academics – felt that many of the proposed questions already had a substantial amount of evidence in the published literature. As such, strong effort is needed to ensure that research evidence is not confined to academic literature, but rather that findings and recommendations are written, translated, and disseminated purposefully and meaningfully to others working in the field of menstrual health, e.g. through educational programmes, through liaison with governments to support legislation. On the other hand, it is possible that those directly involved in the implementation of menstrual health interventions have generated substantial learnings on what the problems are, what interventions work to address them, and how to deliver them effectively in various contexts, but have not formally publishing these learnings. They would thus see those domains as a lower priority despite limited published evidence. More effort is needed to ensure that these learnings are documented, integrated into an evidence base on menstrual health, and equally valued and disseminated across stakeholder groups. Finally, these differences may also reflect variations in what stakeholder groups view as worthy of investment from a limited pool of resources. For example, academics may feel that there is insufficient evidence to invest resources on an intervention, while non-academics may approach such decision-making from a different lens, such as that of human rights.

### Strengths and limitations

This study has several limitations. First, whilst the open invitation along with snowballing technique opened our survey to a wide audience, participants were permitted to self-select, and stringent criteria were not used to evaluate their eligibility for inclusion. As a result, it is possible that the study was affected by non-response bias, and some participants may not have had sufficient expertise to be considered an ‘expert’ in menstrual health. Second, many participants began the surveys in Phases 2 and 3 but did not complete them. The format of the online survey did not allow participants to view the whole document; instead, they had to complete each page before the next page was revealed. This meant participants were unable to decide in advance whether the survey was appropriate or of interest to them until after they had completed their demographic details. A formal analysis of the barriers to completion was not possible, but we hypothesize that the length of the surveys – particularly that used in Phase 3, which had a total of 94 questions each requiring consideration of three criteria – may not have been user-friendly for such a wide audience. This may also have contributed to the high level of consistency in scores across the three criteria. Further, although this was intended to be a global exercise, it was only conducted in English; thus, non-English speaking menstrual health experts may not have been able to participate and, among those who did, some may not speak English as their primary language. This may explain in part the absence of substantial participation from Latin America and the Caribbean and the Middle East, and under-representation from countries in Asia. It is also important to note that the survey required participants to have stable internet connectivity, as it was not available to download; this may also have undermined participation in LMICs. We also note that direct contact was made with well-known academic researchers who were likely to be missed from membership of consortia and acknowledge this was not a systematic capture of all academic researchers internationally. As a result, the findings from this study may not perfectly reflect the opinions of all menstrual health experts. Finally, as noted above, in the interest of ensuring anonymity of the data, it was not possible to confirm that the participants who responded to the survey in Phase 2 were the same as those who responded to the survey in Phase 3. As a result, the perspectives and expertise of the participants may have varied throughout the process.

Nevertheless, this study also has many strengths. While research priorities were previously generated on menstrual health among schoolgirls [3], this is the first study to generate research priorities on menstrual health across the life-course in LMICs. This is particularly timely given the growing momentum among researchers, implementers, and activists in recognizing the importance of menstrual health to female empowerment and gender equity. Additionally, the study utilized a modified CHNRI approach, which is a well-respected and widely utilized systematic approach to research priority setting with transparent criteria. The study also incorporated input from participants representing a wide range of countries, sectors, stakeholder groups, and years of experience in menstrual health. Finally, this study included several sub-analyses to (1) understand the characteristics of those who did and did not complete the surveys and the implications of this for others seeking to use the CHNRI approach to generate research priorities, and (2) to understand how the research priorities differ by stakeholder group and country of origin, along with the implications for knowledge dissemination and translation between academics and non-academics and those in HICs and LMICs.

## Conclusions

In conclusion, this study aimed to generate research priorities on addressing menstrual health across the life-course in LMICs. It identified that the largest number of questions identified as research priorities belong to the domains of ‘understanding the problem’ and ‘designing and implementing interventions’, suggesting that there are still many knowledge gaps in understanding the menstrual experiences of women, girls, and others who menstruate, as well as in understanding the most effective interventions to meet their needs. This study also identified that the top research priorities were not limited to one area of expertise (education, health, WASH, etc.) but were distributed across issues, indicating that research gaps exist in multiple domains of menstrual health which will require collaborative efforts to address. Further, this study identified a strong need to promote equity by understanding the specific menstrual needs and experiences of underserved populations.

As menstrual health continues to gain attention and emphasis as an important component of public health, it is hoped that these research priorities can be utilized by policymakers, programmers, researchers, and funders to guide future research in this area. Recognizing that research priority setting is a dynamic process, it is also hoped that these research priorities will be revisited in an iterative manner as the field continues to evolve.

## Supplementary Material

Supplemental Material

## Paper context

In 2014, research priorities were identified on menstrual hygiene management among schoolgirls in LMICs. This paper identifies an updated set of research priorities for menstrual health across the life-course in LMICs, in consultation with various stakeholders from different geographic regions. The breadth of research priorities indicates the topic remains under-researched and greater evidence is needed for policy and action. Additionally, the different responses between academics and non-academics suggests a need for improved knowledge sharing.
