# Supplementary material for: Research priorities for improving menstrual health across the life-course in low- and middle-income countries
Source: Glob Health Action. 2023 Nov 27;16(1):2279396. doi: 10.1080/16549716.2023.2279396 (PMC10795652; doi:10.1080/16549716.2023.2279396)
Supplement: Supplemental Material [file ZGHA_A_2279396_SM2600.docx]

**SUPPLEMENTARY MATERIALS**

Table S1. Example of a consolidated research question representing similar questions

Table S2. Number of participants that proposed and did not propose research questions for each sub-domain in Phase 2

Table S3. Number of research questions proposed by participants that are highly experienced (7 years or more) and less experienced (6 years or less) in menstrual health in Phase 2

Table S4 Mean number of research priority questions proposed by participants by reported area of expertise

Table S5. Complete list of ranked research questions

**Table S1. Example of a consolidated research question representing similar questions**

| **Consolidated Question** | **Individual questions listed in the survey** |
| --- | --- |
| *What are the effects of female genital cutting on menstrual health?* | ‘What are the effects of female genital cutting on menstrual health’? |
|  | ‘How does FGM/female cutting affect a girl while menstruating and its links to infections’? |
|  | ‘The impact of FGM on menstrual health (it is massive but hidden!)’ |

**Table S2. Number of participants that proposed and did not propose research questions for each sub-domain in Phase 2**

| **Domains and sub-domains** | **Total participants** | |
| --- | --- | --- |
|  | **Proposed questions** | **Did not propose questions** |
| ***Understanding the problem*** |  |  |
| Experiences relating to menstrual health including social context/community attitudes | 78 | 69 |
| Factors affecting menstrual health | 68 | 79 |
| Impact and consequences of poor menstrual health | 73 | 74 |
| ***Designing and implementing interventions*** |  |  |
| Discovery of new interventions | 61 | 86 |
| Developing and testing the effectiveness of interventions and programmes in a controlled trial | 43 | 103 |
| Evaluations of the effectiveness, acceptability, adoption, appropriateness, feasibility, fidelity, cost, coverage, and sustainability of interventions/programmes   - testing the effectiveness of interventions and programmes in real life circumstances | 45 | 102 |
| - evaluations of the cost of interventions and programmes | 34 | 113 |
| - evaluations of the delivery (including acceptability, adoption, appropriateness, feasibility, fidelity, coverage, and reach) of interventions and programmes | 40 | 107 |
| - evaluations on the sustainability of interventions/programmes | 32 | 115 |
| ***Integrating and scale-up*** |  |  |
| Integration of menstrual health interventions and programmes into health, gender, education, WASH, social services | 50 | 97 |
| Scale-up of menstrual health interventions/programmes | 44 | 103 |
| ***Other*** | 28 | 119 |

**Table S3. Mean number of research questions proposed by participants that are highly experienced with 7 years or more, and less experienced (6 years or less) in menstrual health in Phase 2**

|  | Understanding the problem  Mean (sd) | Designing and implementing interventions  Mean (sd) | Integrating and scale-up  Mean (sd) | All  Mean (sd) |
| --- | --- | --- | --- | --- |
| 6 years or less | 5.33 (9.48) | 4.62 (4.42) | 1.49 (1.82) | 11.74 (7.75) |
| 7 years or more | 8.03 (7.10) | 7.10 (7.50) | 2.23 (2.14) | 17.58 (11.93) |
| All | 6.37 (3.72) | 5.57 (5.87) | 1.77 (1.94) | 13.98 (9.92) |
| p value | 0.001 | 0.065 | 0.1 | 0.009 |

Footnote: sd: standard deviation; p value generated from analysis of variance

**Table S4 Mean number of research priority questions proposed by participants by reported area of expertise**

|  | Participants with reported expertise | | | | | | | Participants- no expertise | | | Significance |
| --- | --- | --- | --- | --- | --- | --- | --- | --- | --- | --- | --- |
|  |  | RPQs - means for: | | | | | | RPQs - means for: | | |  |
| Expertise/area of interest | N | Understanding | Design/  development | Integration  /Scale-up | Other | Total all | Total all sd | N | Total all | Total all sd | p value |
| Education | 26 | 5.42 | 4.42 | 1.58 | 0.19 | 11.62 | 7.548 | 56 | 15.09 | 10.74 | 0.142 |
| Gender | 32 | 6.66 | 6.91 | 2.37 | 0.16 | 16.09 | 11.63 | 50 | 12.59 | 8.46 | 0.121 |
| Sexual and Reproductive Health | 51 | 6.2 | 5.56 | 1.96 | 0.2 | 14.02 | 11.446 | 31 | 13.9 | 6.944 | 0.959 |
| Mental Health | 11 | 7.64 | 9.3 | 1.91 | 0.18 | 19.7 | 12.902 | 71 | 13.17 | 9.254 | 0.051 |
| Adolescent Health and Development | 39 | 6.36 | 6.66 | 2.18 | 0.13 | 15.47 | 11.886 | 43 | 12.65 | 7.681 | 0.203 |
| WASH | 35 | 7.89 | 6.91 | 1.77 | 0.2 | 16.77 | 9.72 | 47 | 11.85 | 9.629 | 0.021 |
| Policy | 30 | 6.33 | 4.97 | 2.1 | 0.3 | 13.7 | 8.547 | 52 | 14.14 | 10.718 | 0.849 |

**Table S5. Complete list of ranked research questions**

| **Rank** | **RPS** | **Question** | **Domain** | **Unique ID** |
| --- | --- | --- | --- | --- |
| **1** | 0.956 | What indicators are optimal for assessing menstrual health over time (e.g. related to norms, education, health, rights, etc.)? | Measurement and research | M1 |
| **2** | 0.932 | What are the experiences of girls, women, and others who menstruate in relation to menstrual pain and disorders (e.g. what proportion experience them, what are their perceptions about them, how do they manage them, and what support do they seek and receive for them)? | Understanding the problem | U1 |
| **3** | 0.93 | What new interventions could be developed to address harmful attitudes, norms and stigma and improve communication related to menstruation? | Designing and implementing interventions | D1 |
| **4** | 0.924 | Are girls, women, and others who menstruate able to access and afford their preferred menstrual product/materials; what is the quality of these products/materials; where do they obtain them; and how do they use and dispose of them? | Understanding the problem | U2 |
| **5** | 0.921 | What is the impact of unmet menstrual health needs (e.g. products/materials, WASH infrastructure/services) on the participation and engagement of girls, women, and others who menstruate in school and work and their self-esteem and agency? | Understanding the problem | U3 |
| **6** | 0.919 | What are the experiences and challenges of girls, women, and others who menstruate with particular needs and circumstances (e.g. those living with HIV, those with disabilities, those who are incarcerated, those experiencing homelessness, those who have experienced FGM, trans and gender non-binary persons) in relation to their menstrual health? | Understanding the problem | U4 |
| **7** | 0.915 | What characteristics of menstrual health interventions/programmes enable them to be sustained over time? | Designing and implementing interventions | D2 |
| **7** | 0.915 | How can interventions to address menstrual health (e.g. education, social norm change, distribution of menstrual products/materials, improvements in WASH infrastructure/services, provision of health services for menstrual pain and disorders) be scaled up with quality and equity? | Integrating and scaling up | I1 |
| **9** | 0.913 | What new interventions could be developed to manage menstrual pain? | Designing and implementing interventions | D3 |
| **10** | 0.913 | What are the impacts of unconditional and conditional cash transfer interventions on the menstrual health of girls, women, and others who menstruate, and consequently on their education, work, and social participation? | Designing and implementing interventions | D4 |
| **10** | 0.913 | What are the impacts of providing free/subsidized menstrual products/materials on menstrual health and hygiene? | Designing and implementing interventions | D5 |
| **12** | 0.907 | How can interventions optimally engage boys and men to address harmful attitudes, norms and stigma related to menstruation? | Designing and implementing interventions | D6 |
| **13** | 0.905 | What are the impacts of menstruation-friendly WASH supplies and infrastructure on the menstrual health and hygiene of girls, women, and others who menstruate, and consequently on their education, work, and social participation? | Designing and implementing interventions | D7 |
| **14** | 0.902 | How can information on menstruation be integrated into existing formal and non-formal educational curricula, health services (e.g. contraceptive services, HPV vaccination, FGM support, psychosocial support), social norms, and gender equality interventions/programmes? | Integrating and scaling up | I2 |
| **14** | 0.902 | How can considerations for the menstrual health and hygiene needs of girls, women, and others who menstruate with particular needs and circumstances (e.g. those with disabilities and their caregivers, those living in urban/rural settings, those who have experienced FGM, those in indigenous communities, those in humanitarian settings) be integrated into existing policies and interventions/programmes? | Integrating and scaling up | I3 |
| **16** | 0.901 | How long should menstrual cups be worn, how should they be cleaned between use, and how often should they be replaced? | Designing and implementing interventions | D8 |
| **17** | 0.897 | How can governments integrate menstrual health and hygiene across sectors (e.g. education, health, WASH, gender) and achieve multi-sectoral coordination? | Integrating and scaling up | I4 |
| **18** | 0.895 | How do financial barriers impact the ability of girls, women, and others who menstruate to manage their menstruation? | Understanding the problem | U5 |
| **19** | 0.890 | What tools/instruments/approaches/measures are optimal for assessing the impact of interventions to address menstrual health and hygiene at various programmatic levels (e.g. local, national, global)? | Measurement and research | M2 |
| **20** | 0.889 | How can governments and organizations sustain commitment (e.g. political, financial) to menstrual health and hygiene interventions/programmes? | Designing and implementing interventions | D9 |
| **21** | 0.889 | How can menstrual health and hygiene interventions be optimally tailored for girls, women, and others who menstruate with particular needs and circumstances (e.g. those with disabilities and their caregivers, those living in urban/rural settings, those who have experienced FGM, those in indigenous communities, those in humanitarian settings)? | Designing and implementing interventions | D10 |
| **21** | 0.889 | What are the long term effects (e.g. on health, education, employment, gender equity) of menstrual health and hygiene interventions? | Designing and implementing interventions | D11 |
| **23** | 0.888 | What is the impact of unmet menstrual health and hygiene needs (e.g. information, products/materials, WASH infrastructure/services) the health and wellbeing of on girls, women, and others who menstruate across the life course? | Understanding the problem | U6 |
| **24** | 0.885 | What is the most cost-effective way to finance menstrual health and hygiene interventions/programmes (e.g., out-of-pocket by users, government investment)? | Designing and implementing interventions | D12 |
| **24** | 0.885 | What is the cost and cost-effectiveness of providing various menstrual products and materials for free/at a subsidized rate? | Designing and implementing interventions | D13 |
| **26** | 0.881 | What are the impacts of interventions for parents on communication and support related to menstruation on the menstrual health knowledge and experiences of girls and others who menstruate? | Designing and implementing interventions | D14 |
| **27** | 0.877 | What are the optimal disposal methods for the full range of used menstrual products/materials to ensure human and environmental health and safety? | Designing and implementing interventions | D15 |
| **28** | 0.877 | What new approaches could be used to increase the affordability and accessibility of menstrual products/materials? | Designing and implementing interventions | D16 |
| **29** | 0.876 | What WASH facilities are available in homes, schools, workplaces, health facilities, and communities, and to what extent do these WASH facilities meet the menstrual health and hygiene needs of girls, women, and others who menstruate? | Understanding the problem | U7 |
| **29** | 0.876 | How knowledgeable are men and boys about menstruation, and what role do they play in supporting the menstrual health and hygiene of girls, women, and others who menstruate? | Understanding the problem | U8 |
| **31** | 0.876 | How can the coverage and reach of menstrual health and hygiene interventions be maximized (including to marginalized populations)? | Designing and implementing interventions | D17 |
| **31** | 0.876 | How can integration of menstrual health and hygiene across sectors be optimally monitored and evaluated? | Integrating and scaling up | I5 |
| **31** | 0.876 | How can interventions to address menstrual health and hygiene of girls, women, and others who menstruate with particular needs and circumstances (e.g. those with disabilities and their caregivers, those living in urban/rural settings, those who have experienced FGM, those in indigenous communities, those in humanitarian settings) be scaled up with quality and equity? | Integrating and scaling up | I6 |
| **34** | 0.873 | What is the additional impact of gender-transformative approaches on interventions to improve menstrual health and hygiene? | Designing and implementing interventions | D18 |
| **35** | 0.873 | What national and sub-national policies are in place related to menstrual health and hygiene, and what do they consist | Understanding the problem | U9 |
| **36** | 0.870 | What new disposal methods could be developed to ensure human and environmental health and safety related to disposal of menstrual products/materials? | Designing and implementing interventions | D19 |
| **37** | 0.868 | What is the definition of a meaningful improvement in menstrual health and hygiene, and how can it be measured? | Measurement and research | M3 |
| **38** | 0.868 | Does integration of menstrual health interventions within sexual and reproductive health programming improve their sustainability over time? | Designing and implementing interventions | D20 |
| **38** | 0.868 | How can improvements to WASH facilities/services to meet menstrual health and hygiene needs be sustained over time? | Designing and implementing interventions | D21 |
| **40** | 0.865 | What is the effectiveness of pharmacological and non-pharmacological interventions for management of menstrual pain? | Designing and implementing interventions | D22 |
| **41** | 0.864 | What is the environmental impact of various menstrual products/materials and disposal options? | Understanding the problem | U10 |
| **42** | 0.862 | How does mass media influence menstrual stigma? | Understanding the problem | U11 |
| **43** | 0.861 | What is the impact of health worker training on menstrual health and hygiene on the availability and quality of health services for menstrual health? | Designing and implementing interventions | D23 |
| **44** | 0.860 | What new interventions could be developed to improve menstrual health and hygiene among girls, women, and others who menstruate with particular needs and circumstances (e.g. those with disabilities and their caregivers, those living in urban/rural settings, those who have experienced FGM, those in indigenous communities, those in humanitarian settings)? | Designing and implementing interventions | D24 |
| **45** | 0.859 | What proportion of girls, women, and others who menstruate have correct knowledge about menstruation including signs of abnormality, and what proportion had this knowledge before experiencing menarche? | Understanding the problem | U12 |
| **46** | 0.858 | What is the impact of menstrual pain and/or heavy menstrual bleeding on the social participation of girls, women, and others who menstruate and their ability to function in school and the workplace? | Understanding the problem | U13 |
| **46** | 0.858 | What are the effects of female genital cutting/mutilation (FGC/FGM), particularly Type III, on menstrual health and hygiene? | Understanding the problem | U14 |
| **48** | 0.857 | How can menstrual health and hygiene be addressed from a human rights perspective? | Designing and implementing interventions | D25 |
| **49** | 0.855 | Does inclusion of menstrual health components in health and social service interventions/ programmes increase their attractiveness and acceptability among adolescents and young people? | Designing and implementing interventions | D26 |
| **50** | 0.848 | How does knowledge of and considerations related to menstruation and the menstrual cycle impact the contraceptive choices of girls, women, and others who menstruate? | Understanding the problem | U15 |
| **51** | 0.845 | What interventions to promote menstrual health and hygiene have the greatest impact on the attendance and performance of girls, women, and other people who menstruate in school and work? | Designing and implementing interventions | D27 |
| **52** | 0.842 | How can key stakeholders (e.g. employers or school officials) be optimally engaged to address menstrual health and hygiene and held accountable for doing so? | Integrating and scaling up | I7 |
| **52** | 0.842 | How can menstruation-friendly WASH standards be incorporated into broader standards, such as for building codes for WinS? | Integrating and scaling up | I8 |
| **54** | 0.839 | How do stress and anxiety associated with menstruation affect the menstrual experiences of girls, women, and others who menstruate? | Understanding the problem | U16 |
| **55** | 0.838 | Which standards need to be maintained in order to ensure quality and equity in the scale up of menstrual health and hygiene interventions/programmes? | Integrating and scaling up | I9 |
| **56** | 0.836 | What is the impact of parents’/caregivers’ lack of knowledge and support for menstrual health and hygiene on the ability of girls and others who menstruate to manage their menstruation? | Understanding the problem | U17 |
| **57** | 0.836 | How are teachers prepared to teach students about menstruation, and what is the impact of this on their ability to provide education related to menstrual health and hygiene? | Understanding the problem | U18 |
| **57** | 0.836 | Do employers incorporate attention to menstrual health and hygiene in formal and informal workplace environments, and what is the impact of this on the participation and engagement of women, girls, and others who menstruate at work? | Understanding the problem | U19 |
| **59** | 0.833 | How can the feasibility and acceptability of environmentally-friendly menstrual products/materials (including those that are reusable) be improved? | Designing and implementing interventions | D28 |
| **59** | 0.833 | How can civil society organizations increase and sustain government attention to and engagement in menstrual health and hygiene? | Integrating and scaling up | I10 |
| **59** | 0.833 | What are the experiences of women and others who menstruate in relation to menopause (e.g. what knowledge and attitudes do they have about it, how do they manage it, what support do they seek and receive)? | Understanding the problem | U20 |
| **59** | 0.833 | What guidance and support networks exist for caregivers of a person with a disability to manage their menstrual health and hygiene? | Understanding the problem | U21 |
| **63** | 0.825 | Does engagement of men and boys increase the impact of interventions on menstrual health and hygiene? | Designing and implementing interventions | D29 |
| **64** | 0.825 | What is the feasibility of government financing for the provision of free/subsidized menstrual products/materials? | Designing and implementing interventions | D30 |
| **65** | 0.821 | What are the most effective interventions to promote menstrual health and hygiene at various points in the life course? | Designing and implementing interventions | D31 |
| **66** | 0.821 | Can the integration of menstrual health and hygiene interventions with sexual and reproductive health and rights interventions result in cost-savings for intervention delivery, and in broader cost-effectiveness? | Designing and implementing interventions | D32 |
| **67** | 0.818 | What is the economic impact of purchasing menstrual health and hygiene supplies (e.g. products/materials, pain medication) on individual and family finances? | Understanding the problem | U22 |
| **68** | 0.816 | What are the environmental costs of the full range of menstrual products/materials? | Designing and implementing interventions | D33 |
| **69** | 0.816 | Can menstrual health and hygiene be used as an entry point to improve the acceptability of sexual and reproductive health or comprehensive sexuality education, and vice versa? | Designing and implementing interventions | D34 |
| **70** | 0.812 | What are the most appropriate and acceptable menstrual products/materials for different populations and settings? | Designing and implementing interventions | D35 |
| **71** | 0.803 | What is the minimum ‘dosage’ of menstrual health and hygiene educational interventions required to improve knowledge and practices related to menstrual health and hygiene? | Designing and implementing interventions | D36 |
| **72** | 0.802 | How do menstrual cycle predictor cell phone apps influence the menstrual experiences of girls, women, and others who menstruate? | Designing and implementing interventions | D37 |
| **73** | 0.795 | What is the feasibility and acceptability of menstrual-friendly policies in schools and in workplaces? | Designing and implementing interventions | D38 |
| **74** | 0.795 | How can menstrual health and hygiene interventions/programmes be delivered with fidelity over time? | Designing and implementing interventions | D39 |
| **75** | 0.794 | What are the impacts of regulations/standards for menstrual products/materials on menstrual health and hygiene? | Designing and implementing interventions | D40 |
| **76** | 0.793 | What new interventions could be developed to support menstrual health and hygiene needs in the workplace? | Designing and implementing interventions | D41 |
| **77** | 0.790 | What are the impacts of social norm change interventions at the community level (including mass media) on the menstrual health and hygiene of girls, women, and others who menstruate? | Designing and implementing interventions | D42 |
| **78** | 0.785 | How can lessons learned from the delivery of menstrual health and hygiene interventions be optimally documented and shared? | Measurement and research | M4 |
| **79** | 0.783 | What new technologies could be developed for collecting/absorbing and cleaning menstrual blood? | Designing and implementing interventions | D43 |
| **80** | 0.782 | What policies are effective in improving menstrual health and hygiene, and what approaches are most effective in strengthening the implementation of such policies? | Designing and implementing interventions | D44 |
| **81** | 0.778 | What is the impact of comprehensive sexuality education on students’ knowledge about menstruation? | Designing and implementing interventions | D45 |
| **82** | 0.774 | Which personnel are most acceptable for the delivery of school and community-based interventions on menstrual health and hygiene? | Designing and implementing interventions | D46 |
| **83** | 0.761 | What is the role of various stakeholders (e.g. government, NGOs, etc.) in the scale up of menstrual health and hygiene interventions/programmes? | Integrating and scaling up | I11 |
| **84** | 0.754 | What is the relationship between menstrual health and hygiene and parasitic diseases (e.g. female genital schistosomiasis)? | Understanding the problem | U23 |
| **85** | 0.753 | What frameworks or theories (e.g. behavioural theory) can be leveraged to design menstrual health interventions? | Designing and implementing interventions | D47 |
| **86** | 0.752 | What is the impact of local production of reusable menstrual products/materials on their cost? | Designing and implementing interventions | D48 |
| **87** | 0.735 | What role can national and district-level governments play in measuring the return on investment from menstrual health and hygiene interventions/programmes? | Designing and implementing interventions | D49 |
| **88** | 0.730 | What are the most effective interventions to promote menstrual health and hygiene among girls, women, and other menstruators with particular needs and circumstances (e.g. those with disabilities and their caregivers, those living in urban/rural settings, those who have experienced FGM, those in indigenous communities, those in humanitarian settings)? | Designing and implementing interventions | D50 |
| **89** | 0.724 | What support is needed to encourage relevant, timely, and rigorous research on menstrual health and hygiene, and which actors need to be engaged in the research process? | Measurement and research | M5 |
| **90** | 0.717 | What new approaches, curricula, and/or learning materials could be developed to improve education on menstrual health and hygiene? | Designing and implementing interventions | D51 |
| **91** | 0.706 | How do community attitudes and beliefs influence understandings and responses to menstrual pain? | Understanding the problem | U24 |
| **92** | 0.701 | What kind of information and support for menstrual health and hygiene are health workers willing and able to provide? | Understanding the problem | U25 |
| **93** | 0.693 | What new interventions could be developed to improve the quality of health services for menstrual health? | Designing and implementing interventions | D52 |
| **94** | 0.667 | What is the impact of interventions to improve menstrual health and hygiene on the sexual and reproductive health (e.g. use of contraception, adolescent pregnancy, detection of cervical cancer, reproductive tract infections) of girls, women, and others who menstruate? | Designing and implementing interventions | D53 |
